# e-Health: A Future Solution for Optimized Management of Elderly Patients. GER-e-TEC™ Project

**DOI:** 10.3390/medicines7080041

**Published:** 2020-07-23

**Authors:** Abrar-Ahmad Zulfiqar, Noël Lorenzo-Villalba, Oumair-Ahmad Zulfiqar, Mohamed Hajjam, Quentin Courbon, Lucie Esteoulle, Bernard Geny, Samy Talha, Dominique Letourneau, Jawad Hajjam, Sylvie Erve, Amir Hajjam El Hassani, Emmanuel Andres

**Affiliations:** 1 Diabètes et Maladies Métaboliques, Service de Médecine Interne, Hôpitaux Universitaires de Strasbourg, 67000 Strasbourg, France; noellorenzo@gmail.com (N.L.-V.); emmanuel.andres@chru-strasbourg.fr (E.A.); 2 Engineer Department, Neoma Business School, 51100 Reims, France; oumair.zulfiqar.19@neoma-bs.com; 3 Predimed Technology, 67300 Schiltigheim, France; mohamed.hajjam@predimed-technology.com (M.H.); quentin.courbon@predimed-technology.com (Q.C.); esteoullelucie@gmail.com (L.E.); 4 Service de Physiologie et Laboratoire d’Explorations Fonctionnelles, Hôpitaux Universitaires de Strasbourg, 67000 Strasbourg, France; bernard.geny@chru-strasbourg.fr (B.G.); samy.talha@chru-strasbourg.fr (S.T.); 5 Equipe de recherche EA 3072 “Mitochondrie, Stress oxydant et Protection musculaire”, Faculté de Médecine de Strasbourg, Université de Strasbourg, 67000 Strasbourg, France; 6 Fondation de l’Avenir pour la Recherche Médicale Appliquée, 75015 Paris, France; dominique.letourneau@u-pec.fr; 7 Centre d’Expertise des TIC pour l’autonomie (CenTich) et Mutualité Française Anjou-Mayenne (MFAM)–Angers, 49000 Angers, France; jawad.hajjam@centich.fr (J.H.); sylvie.erve@centich.fr (S.E.); 8 Laboratoire IRTES-SeT, Université de Technologie de Belfort-Montbéliard (UTBM), Belfort-Montbéliard, 90000 Belfort, France; amir.hajjam@utbm.fr

**Keywords:** telemedicine, remote monitoring, GER-e-TEC, elderly patient, artificial intelligence, geriatric syndromes, detection of the precursory signs of decompensation of geriatric syndromes

## Abstract

**Background:** Elderly residents in nursing homes have multiple comorbidities (including cognitive and psycho-behavioral pathologies, malnutrition, heart failure, diabetes, chronic obstructive pulmonary disease, and renal failure) and use multiple medications. **Methods:** The GER-e-TEC project aims to provide these fragile and complex patients with telemedicine tools, more specifically telemonitoring, backed by a well-defined and personalized protocol. **Results:** Medically, this implies the need for regular monitoring and a high level of medical and multidisciplinary expertise for the healthcare team. The tools use non-invasive communicating sensors and artificial intelligence techniques, allowing daily monitoring with the ability to detect any abnormal changes in the patient’s condition early. **Conclusions:** The GER-e-TEC project specifically considers the challenges of aging residents and significant challenges in nursing homes, with the main geriatric syndromes (falls, malnutrition, cognitive-behavioral disorders, and iatrogenic conditions).

## 1. Introduction

Life expectancy continues to rise worldwide, approaching or exceeding 85 years for women and 80 years for men in Europe [1]. In 2060, one-third of the French population will be over 60, with 5 million over 85 compared to 1.4 million today. This epidemiological trend will require more healthcare expenditures and a rebalance of public finances.

At the end of 2015, the number of persons in nursing homes reached 728,000 in France according to the Survey of Establishments for Elderly People (EHPA) implemented by the Directory of Research, Evaluation, and Statistics Studies (DRESS) [2]. Older people enter nursing homes later in their lives, and when they do, they are most often dependent, as shown in the study carried out in 2011 by Morley. In that study, the proportion of residents classified from 4 to 1 in the Iso-Resource Group (GIR) represented 91% of cases [3].

Today, residents in nursing homes have multiple comorbidities (such as heart failure, diabetes, chronic obstructive pulmonary disease (COPD), and renal failure) and are on multiple medications. From the medical perspective, this implies the need for regular monitoring and a high level of medical expertise. In addition, people aged 75 and older often have a certain level of fragility, determined by a reduction in functional reserve of vital organs with age, with geriatric syndromes which are specific to the age group (malnutrition, sedentary lifestyle).

According to the results of a national analysis carried out by the National Agency for the Evaluation and Quality of Social and Medico-Social Establishments and Services (ANESM) in 2015, one out of every two residents in nursing homes are admitted to a hospital each year for an average hospital stay of three weeks. Moreover, these hospitalizations are frequently preceded by an emergency department admission [4]. Thus, visits to the emergency room are frequent for residents in nursing homes, with one in four patients subsequently admitted at least once per year and one in ten residents admitted at least twice per year [5]. According to France’s national High Committee for the Future of Health Insurance (HCAAM), hospitalizations of this population represent an expenditure of 1.7 billion euros per year for health insurance [6].

Urgent hospitalizations for residents in nursing homes could be preventable in up to 67% of cases [7]. High rates could be reduced by improving decision-making procedures. Among the causes of preventable hospitalizations is the lack of medical availability or lack of communication between paramedics and healthcare physicians [8].

Telemedicine, particularly telemonitoring, has been shown to be effective in the management of chronic pathologies such as heart failure. For example, the MyPredi remote monitoring platform, formerly known as E-care, has been tested for monitoring chronic diseases, both in hospitals and at home. The system makes it possible to combine the data collected daily with the operator’s clinical knowledge of the patient using artificial intelligence tools for personalized and predictive monitoring. These systems are particularly suited for monitoring patients in nursing homes and their geriatric risks. As a result, the GER-e-TEC program was created and will be detailed in the present article.

## 2. Some Examples of Remote Monitoring Programs in Elderly Individuals

According to the French Public Health Code, telemedicine is defined as a form of remote medical practice using information and communication technologies. It connects different networks with a patient and one or more health professionals, and if necessary, other professionals providing care to the patient. Telemedicine, and specifically telemonitoring, has shown its effectiveness in the follow-up of chronic diseases such as heart failure and hypertension [9]. Monitoring patients with chronic pathologies using telemedicine systems is one way of optimizing the cost of care for these patients. Telesurveillance aims to give autonomy at home to people suffering from various pathologies and handicaps which would normally compel them to hospitalization or placement in specialized institutions: patients suffering from certain chronic diseases, disabled, but also dependent elderly people [10].

The telemonitoring objectives are ambitious: improvement in morbidity and mortality, reduction in rehospitalizations, and improvement in the quality of life and in medico-economic costs. Remote monitoring is a medical procedure that results from the transmission and interpretation by a doctor of a clinical, radiological, or biological indicator, collected by the patient or by a health professional [11]. The interpretation of the data can lead to the decision of early medical intervention. The data are analyzed by a doctor, who could delegate management to another health professional from a task validated by the Regional Agency of Health. Management decisions are based on a written protocol for monitoring the indicator that has been validated by the attending physician or a designated responsible physician. This system, therefore, allows the attending physician to know whether the patient is stable and warn about the possible destabilization of the indicator, which could be immediately corrected to prevent complications leading to hospitalization. The advantages of medical telemonitoring include an accumulated prevention of conditions posing a risk of acute decompensation of chronic pathology with consequently a better quality of life, a reduction in rehospitalizations and their economic cost, better medical follow-up, and better commitment and compliance of the patient to the management of diseases with, consequently, better therapeutic compliance [12].

The digitized selected indicators are available to health professionals for medical interpretation through different methods (via different internet media and communication channels, such as web-based, mobile apps, and direct sensor connection to the internet/cloud application). This must be the location of the patient’s primary medical file so that a change in a clinical indicator is noted and the prescription registered. These actions must be traced and can be recorded in the shared medical file. The transfer of these indicators must be secured. Integrating telemonitoring into the care of a patient with a chronic disease can help prevent complications and unscheduled hospitalizations.

All the benefits described above strongly support the deployment of telemonitoring and teleconsultation in the elderly, and in particular, in dependent patients. In the area of heart failure, the rate of hospital referrals for patients over the age of 70 has increased by 30% in the past ten years [12]. In Quebec, remote monitoring of patients with heart failure at home has reduced the rate of hospitalization by more than 60% [12].

EPI-MEDIC [13] is a European project implemented in France, Italy, and Sweden. It has allowed for the development of a personal ECG monitor (PEM) for the early detection of cardiac ischemia and arrhythmia. A clinical study was conducted with 794 patients, in which healthcare professionals performed 1372 PEM ECGs. In addition, 188 patients in self-care situations performed 1287 ECGs with their PEMs. The EPI-MEDIC system generates different levels of alerts and transmits these alerts with the patient’s ECG results and personal electronic medical record to healthcare providers by way of a mobile phone. In severe cases, the alerts are automatically sent to the nearest emergency call center. Clinical trials have shown that the EPI-MEDIC solution can save lives and is invaluable for prehospital triage.

Developed in England, TeleHealth [14] is a remote monitoring platform that monitors a patient’s vital signs and activity. It is designed for elderly people who live alone and suffer from chronic diseases (congestive heart failure and chronic obstructive pulmonary disease). A primary study was conducted with 36 participants for a duration of up to one year, depending on the patient. Each day, the patients measured their vital signs (blood pressure and weight for CHF, oxygen saturation level for COPD) with sensors, as well as their activity (passive infrared motion sensor and/or a chair or bed sensor). An algorithm is used to process the data and sends an alert in case of an abnormality. Measurements and alerts are sent to the platform wirelessly and can be consulted online by both the patient and the physician. TeleHealth allows for improved prevention and faster medical interventions, and consequently, an overall increase in patient wellbeing

Two other projects were conducted for the remote monitoring of heart failure: the Doctor/Patient Health Interactive Platform [15] and SEDIC [16] studies. The SEDIC study showed a significant decrease in cardiovascular mortality, and the results of the first mentioned study are underway.

The COMPAS [17] and ECOST [18] studies focused on pacemaker monitoring. These studies demonstrated the safety and benefit of cardiac telemonitoring in randomized studies of 538 and 433 patients, respectively. The COMPAS study showed a 56% reduction in follow-up consultations and a reduction in hospitalizations for atrial arrhythmia or stroke. The ECOST study showed that telemonitoring of implantable defibrillators can have a positive medical-economic impact, including a reduction in inappropriate shocks due to early regulations and a reduction in hospitalizations related to these shocks.

Dary et al. conducted a study on the telemonitoring of atrial fibrillation. In total, 200 patients were enrolled at the start of the study: 45% male and 55% female, with an average age of 67 years; 16% of the enrolled patients were over the age of 80; 35% had a history of treated arrhythmias. For the 200 patients enrolled in the study, 63 had a known arrhythmia and 137 patients were in sinus rhythm at the outset. Of the latter group, 61 (45%) maintained a normal pattern, 43 (22%) had a detected atrial fibrillation, and 33 (16%) had episodes of tachycardia. For the 63 patients in arrhythmia, 24 had their rhythm monitored and 39 had their heart rate monitored. In total, for 33% of this study’s patients, telemonitoring improved the diagnosis and treatment of atrial fibrillation, allowing their treatment to be adapted according to rhythm, rate, and conduction time [19].

In 2003, Goldberg et al. [20] published the WHARF study, which included the largest randomized multicenter sample and compared the value of telemonitoring aftercare to that of in-person aftercare. After six months, there was no significant difference in the rate or time frame of hospital readmissions (*p* = 0.28), but there was a reduction in mortality rates (*p* < 0.003). The Tele-HF study (Telemonitoring to Improve Heart Failure Outcomes) [21] included patients who had presented with decompensated heart failure in the last 30 days. The average age was 61. There was no notable difference between the two groups based on the criteria of mortality rate and hospitalization for any cause, nor on the secondary criteria of death, hospital readmission, and length of hospital stay.

Other projects have focused on medical telemonitoring. Minutolo et al., used a decision support system for remote monitoring of people with heart failure [22]. The program is based on systems grouping the data related to the patient: posture, cardiac sensor, physical activities, and alerts.

The SETAM study (Strategy of Early Detection and Active Management of Supraventricular Arrhythmia with Telecardiology) was a randomized study of telemedicine in rhythmology, looking at two groups of patients with pacemakers. It showed that telemedicine promoted the early detection of events, with a 66% reduction in hospitalizations related to atrial arrhythmias, and the prevention of strokes. The study showed that telecardiology allowed for earlier diagnosis and treatment of patients with atrial arrhythmias, and a reduction in the atrial fibrillation burden after nine months of monitoring in patients in sinus rhythm upon enrollment, with a score of CHA2DS2-VASc >= 2. In total, 595 patients were enrolled in 57 general hospitals (CHGs) in France, for an average period of 12.8 +/− 3.3 months and an average age of 79 +/− 8 years [23].

Many studies have been conducted in the field of activity monitoring for the elderly, in particular to prevent the risk of falls. The majority of the devices are designed for home care, but they are also suitable for monitoring in nursing homes.

One such device was developed by Gerhome [24], which seeks to help elderly people to continue to live at home. Sensors are placed in various parts of the home and communicate wirelessly (by radio frequency) with a computer center. These sensors include ambient sensors (temperature, humidity, and luminosity), volumetric sensors, occupancy sensors (bed, armchair), water sensors, electricity sensors, door sensors, and image sensors.

An experiment conducted in a nursing home in Antibes highlighted the effectiveness of activity detection devices in nursing homes. For example, if a resident gets up during the night, an alert is sent to the night nurse who can subsequently take all the necessary precautions for a potential fall. RESPECT [25] has developed an innovative telehealth device, a smart insole that communicates wirelessly and measures activity and energy levels autonomously. The insole helps with physical activity and the monitoring of frailty indicators, in particular those related to walking. It measures weight, the number of strides, total distance, and the average walking speed. Its battery life is 7 to 13 months. Several tests have demonstrated the accuracy of the technology of smart insoles. AphyCare Technologies has developed an alert bracelet called Séréo’Z [26] to address the risk of falling. Séréo’Z is a multi-sensor ambulatory monitoring system that is worn on the wrist. It collects and processes physiological and activity data (breathing rate, heart rate, skin temperature) and detects falls. The data are transmitted via a secure radio link to a remote transmitter connected to a telephone socket in the home. Alerts are sent to a remote assistance center. A similar bracelet, Vivago [27], has been developed in Finland.

In France, the Limoges University Hospital began the Icare project [28]. Unique to Europe, this pilot research project assesses the effectiveness of home medical telemonitoring for elderly people with chronic diseases. The aim is to prevent the loss of autonomy of elderly individuals at home and to show that remote monitoring of chronic diseases in elderly patients avoids decompensation (disruption of balance) and unscheduled hospitalizations. The study is being conducted over a 12-month period with 500 elderly volunteers. Some patients will utilize remote monitoring using biometric sensors installed in their homes. These sensors (not worn) monitor vital parameters, such as blood pressure, blood sugar, weight, blood oxygen saturation, or temperature. A box transmits these data daily and securely to the attending physician and the private nurse, who usually follow the patient simultaneously, but also to the expert geriatrician in the University Hospital.

The study by Edirippulige et al. suggested that the quality of “medical evidence” for telemedicine is low, but this should be considered carefully since there is only one randomized study [29]. In addition, most of the studies are observational and qualitative. They mainly result from surveys and interviews (patients and healthcare personnel). The study showed that there is evidence of the feasibility and satisfaction of stakeholders in the use of telemedicine in long-term care facilities in several clinical specialties and especially, geriatrics [29]. With respect to costs, this study highlighted the following observations: (i) the costs related to the medical coordination of patients followed up by telemedicine decreases when there are more than 850 uses per year; (ii) for dermatology, remote expertise makes telemedicine profitable; (iii) for geriatrics, it is especially the geriatric assessment and patient education which appear to be the most profitable.

However, in all situations, telemedicine seems likely to help optimize the care and cost to patients treated by avoiding certain emergencies and repetitive hospitalizations [30].

## 3. MyPredi: A Platform for Early Detection and Reporting of Risk Situations

The MyPredi remote monitoring platform, formerly known as the E-care project, selected within the framework of the Investments for the Future call for projects known as “Health and autonomy in the living environment thanks to digital technology”, has the initial main objective of optimizing the follow-up of patients with heart failure by detecting the warning signs of cardiac decompensation through a telemedicine system that combines motivation and education tools [31]. The project will, in theory, make it possible to decrease the number of rehospitalizations, reduce the length of hospitalization, and improve the quality of life of these patients.

It helps the medical professionals by automating the processing of information from sensors by the automatic generation of alerts, in order to detect early indicators of cardiac decompensation [32]. This analysis is adapted to the phenotype of each patient (personalized medicine). The platform also allows the sharing of heterogeneous knowledge to integrate the information necessary for monitoring any medical condition (predictive medicine).

Early detection of cardiac decompensation involves integrating data from sensors: weight, blood pressure, heart rate, oxygen saturation, patient ergonomics, and questionnaires asking the patient about the symptoms of cardiac decompensation (edema, dyspnea, habitual fatigue), as well as the addition of dietary monitoring. All these consolidated elements, along with the patient profile [12], allow detection of cardiac anomalies with the goal of preventing situations at risk of cardiac decompensation (preventive medicine).

The MyPredi platform uses a system to define a controlled vocabulary (diseases, drugs, symptoms, etc.) and to model the concepts related to the monitoring of heart failure [32]. The effectiveness of the system for diagnostic purposes implies that operational semantics are included, semantics which specify how the knowledge modeled in the system will be processed and automatically produce further knowledge. The reasoning part is based on an inference engine where the rules are either introduced by medical experts or from generated data, then, validated by medical experts.

**Figure 1:**
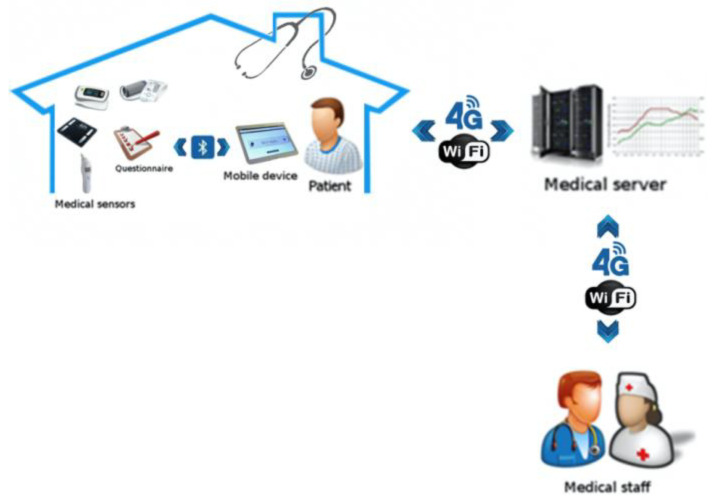
Remote monitoring according to the MyPredi platform. (Source: PrediMed, Schiltigheim, France).

As shown in Figure 1, the MyPredi platform includes a patient module and a server module. The first is installed in the patient’s residence, making it possible to collect and integrate data from different types of non-invasive medical sensors [32] or exchanges in the form of questions and answers. All of the functionalities are embedded in a mobile device, such as a tablet, in order to allow greater patient autonomy. The server module centralizes the data from the patient module and the interpreters. The two modules communicate securely with each other according to standards used in healthcare informatics including Health Level 7 (HL7 V2.6 associated to IHE DEC PCD-01 (patient care device)) and Integrating the Healthcare Enterprise (IHE) protocols.

## 4. The Patient Module

This module, installed in the patient’s house, includes several sensors (devices, also called Agents) and a receiver (Aggregation Manager) for collecting data from physiological sensors (thermometer, oximeter, blood pressure monitor, scale, etc.) as well as exchanges in the form of questions and answers. The receiver (computer, touch pad, or smartphone) sends the data to the server via the internet. In the MyPredi platform, touch pads are used with an ergonomic application that allows the patient or their relatives to submit their measurements for advice and to communicate directly with their doctor.

## 5. The Server Module

The server receives data from all patient modules and manages authentication and communications security, as well as data consistency. These are stored to preserve the privacy of patients. The server uses systems and an intelligent component to detect risk situations and warn caregivers.

A service portal is used to allow users, depending on their role (patient, caregiver, relatives, etc.), to access and follow the evolution of vital signs as well as the general condition of the patient or even to enter medical information and consult transmitted alerts.

The MyPredi platform was initially tested in a 20-bed Internal Medicine unit receiving patients from emergency facilities as part of a care system for patients with heart failure (HF) at the University Hospitals of Strasbourg [33]. In this study, 180 patients were included and 1500 measurements were made. The patient population profile included in this study was elderly patients, multiple medical diagnoses in more than 90% of the cases, and loss of autonomy in 25%. Healthcare professionals used the E-care platform daily. This experiment made it possible to validate the technological choices, to consolidate the system, and to test the robustness of the platform. An analysis shows the relevance of the alerts generated (indicators of deterioration of the patient’s heart condition).

**Figure 2:**
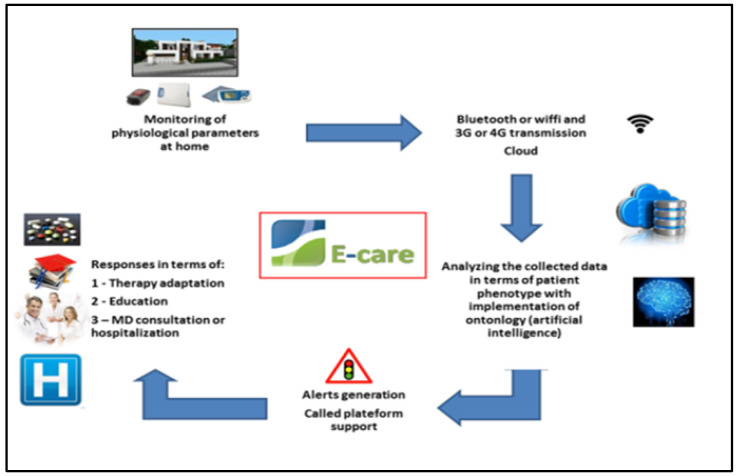
Structure of telemedical platform developed in the E-care project. (Source: PrediMed, Schiltigheim, France).

The MyPredi platform allows automated, non-invasive generation of alerts related to the detection of situations at risk of cardiac decompensation, subsequently preventing hospitalization. Currently, an upgraded telemonitoring platform has been deployed in the homes of patients with heart failure in the Bas-Rhin as part of the PRADO INCADO project (project funded by ARS of Alsace) [33] (Figure 2).

## 6. Development of the MyPredi Platform

The MyPredi platform integrates the monitoring of other chronic diseases including diabetes, hypertension, and COPD [34]. The MyPredi platform is an evolutive platform with original architecture and proven functionalities to manage patients with chronic pathologies who require long-term follow-up. It consists of a patient module, with a tablet and sensors, and a server module, which receives and processes the data collected from the various patient modules. The server module combines semantic web and artificial intelligence technologies (AI). An inference engine is used to monitor the patient’s health and to detect any abnormal situation [12]. We propose a medical telemonitoring platform with an open and flexible architecture, offering the ability to build predictive models of risk situations.

In the detection of anomalies, we use an expert system based on inference rules built with medical experts. These rules are generic and evolve with the new knowledge generated by deep learning algorithms on the data collected and the evolution of the patient’s condition. All alerts detected by the system are forwarded to the nursing staff responsible for monitoring the patient.

In the future, the MyPredi platform should gradually be enriched with other communicating sensors such as the ECG and electronic stethoscope, integrating the signal processing tools that will enable better detection of situations of risk [34,35]. Other communicating sensors could also be considered, such as an electronic spirometer, in order to complete the platform and extend its interest in other chronic diseases, such as asthma, COPD, and chronic renal failure [36,37].

## 7. Geriatrics and e-Technology: GER-e-TEC Project

We have implemented the GER-e-TEC project incorporating all the information mentioned above. The aim of the project is to study the contribution of telesurveillance to residents in nursing homes of Strasbourg University Hospitals through a structured and a protocolized medical follow-up to avoid acute decompensations and complications of geriatric conditions. The project relies on partners who work together, a multidisciplinary team consistent with the requirements in terms of medical, scientific, and structural skills.

### 7.1. Objective

The objective of our work is to develop a codified preventive approach for the management of the main geriatric risks in nursing homes using a personalized remote monitoring platform of residents, in order to avoid factors leading to acute decompensation in the elderly.

The collection of information by the platform allows not only personalized monitoring, but also a better understanding of the patient, providing a particularly effective tool for information transmission between the medical staff (doctors, nurses) in nursing homes. This data collection will also permit the identification of markers subsequently used to improve the early detection of decompensation, thus, improving the monitoring of patients and hopefully reducing the number of hospitalizations. This work creates a resident liaison file.

### 7.2. Main Developments and Functions

The platform used helps caregivers by automating the processing of sensor information, questions, and questionnaires in order to detect and report medical risk situations early. MyPredi will provide personalized care for the main geriatric risks to avoid the occurrence of acute factors leading to decompensation in elderly patients. The information collected will be supplemented by codified therapeutic care, according to international recommendations in nursing homes.

**Figure 3:**
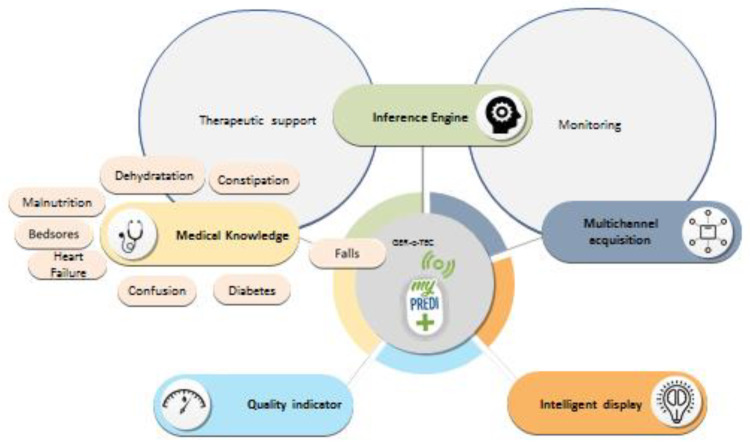
GER-e-TEC project. (Source: PrediMed, Schiltigheim, France).

This platform provides all paramedical and medical health professionals with information on the resident’s geriatric data. This information will be updated regularly, including anthropometric, nutritional, cognitive, and iatrogenic data. Together, the data will provide a real-time complete picture integrated into an electronic platform, creating a standardized gerontological assessment based on simple and rapid measures. Geriatric risks include the risks of falling, constipation, dehydration, confusion, iatrogenic, malnutrition, heart failure, diabetes, infections, and bedsores (Figure 3).

The platform uses an intelligent algorithm to process data and generate alerts based on medical knowledge of the diseases treated, as modeled by ontologies. The general principle adopted by this platform is the anticipation of decompensation through the detection of warning signs that lead, ultimately, to hospitalization. A series of measures and questionnaires will be integrated into the platform for adaptive and personalized monitoring of the patient’s state of health in nursing homes. This study benefited from the support of CENTICH (National Expertise Center for Information and Communication Technologies for Autonomy).

### 7.3. Expected Results and Prospects

The improvements and perspectives of this project will be, in the medium-term:

To decrease the number of preventable hospitalizations by implementing the platform in nursing homes;

To ensure continuity and regularity in the follow-up of patients, particularly in those with complex chronic diseases;

To improve the quality of life of nursing home patients through better follow-up;

To take into consideration the residents’ needs and expectations;

To improve access to hospital care facilities, reducing the use of emergency services, medical care, inappropriate hospitalization, and transport; to promote safe medical practices, sharing, and optimization of knowledge in order to safely improve information exchange among health professionals to better articulate the different healthcare levels.

### 7.4. Project Stages

The three stages of the project are:

To deploy the system at the residents’ bedside in nursing homes;

To validate the solution and develop it with healthcare professionals;

To validate the alerts.

The phasing of the GER-e-TEC project involves two stages:

Phase 1: this corresponds to the implementation and validation of technological processes in an Internal Medicine-Geriatrics department at Strasbourg University Hospitals in the period June 2019–November 2019 (preliminary study (evidence concept));

Phase 2: this aims at generalizing the telemonitoring of elderly patients in nursing homes related to Strasbourg University Hospitals as the referral medical facility (study itself).

### 7.5. Methodology

**Figure 4:**
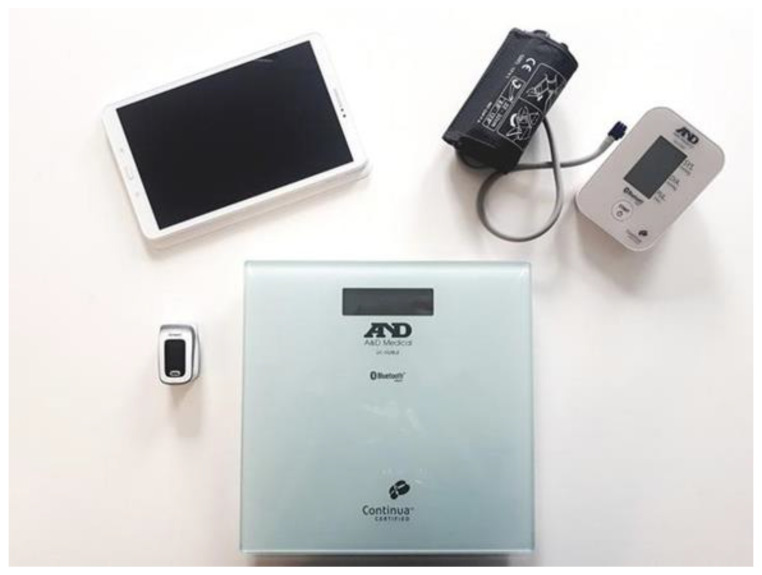
Devices used within the framework of the MyPredi platform. (Source: PrediMed, Schiltigheim, France).

Using a tablet and connected sensors (Figure 4), the patient’s vital signs are measured daily: blood pressure, heart rate, weight, oxygen saturation, capillary glucose, temperature, and physical activity. The technical characteristics of the sensors are detailed in Table 1. The entire device of the MyPredi platform is ISO13485 medical certified with CE marking.

The vital signs are automatically sent to the MyPredi platform. Questionnaires are also integrated in order to be regularly updated by the medical and paramedical team. This includes the main gerontological risks and disorders such as constipation (frequency of daily bowel movements), hydration (quantification of daily fluid intake), iatrogenic risk, heart failure, quality of sleep (neurological, psycho-behavioral), and level of bed rest (bedsores/physical activity).

**Figure 5:**
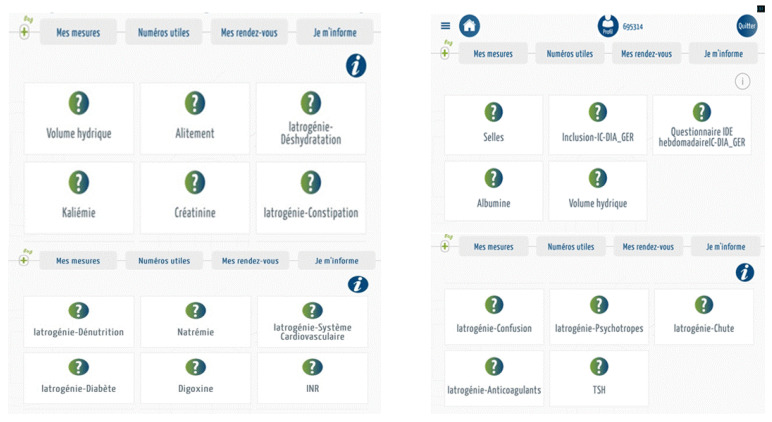
Description of the MyPredi gerontological risk dashboard. (Source: PrediMed, Schiltigheim, France).

Figure 5 illustrates the dashboard of geriatric risks available on the tablet of caregivers in charge of nursing home residents.

**Figure 6:**
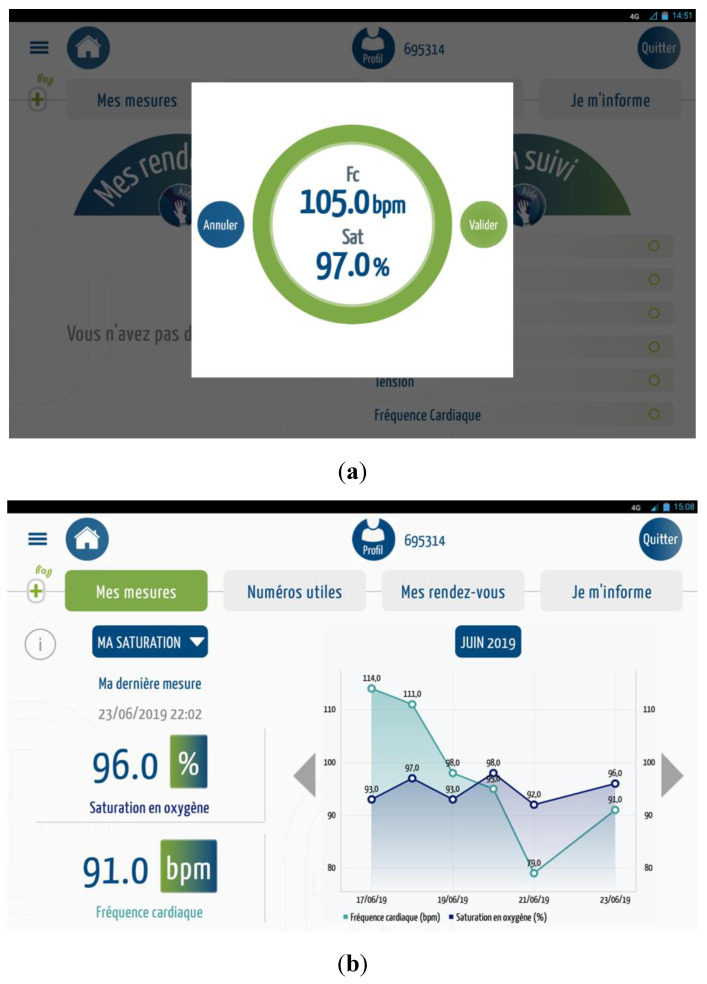

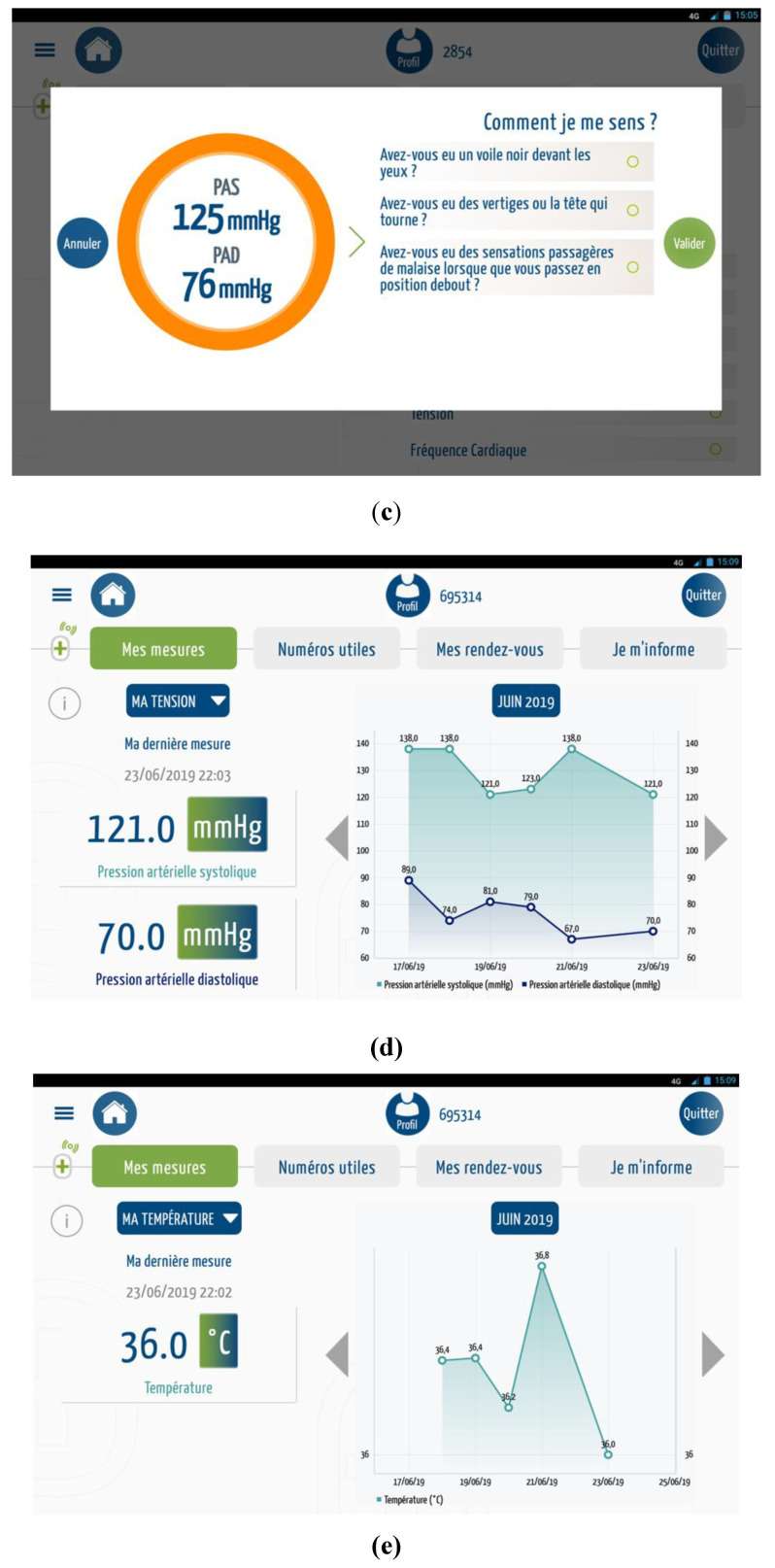

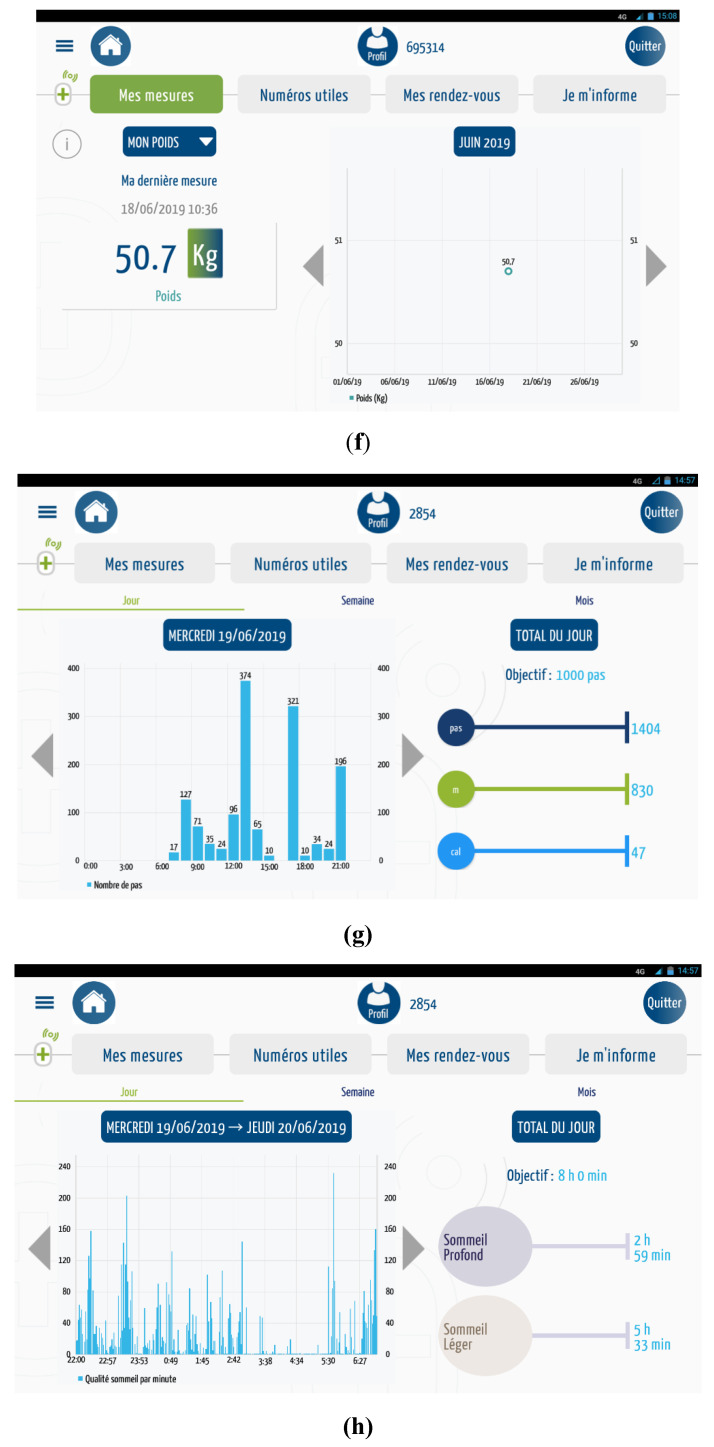

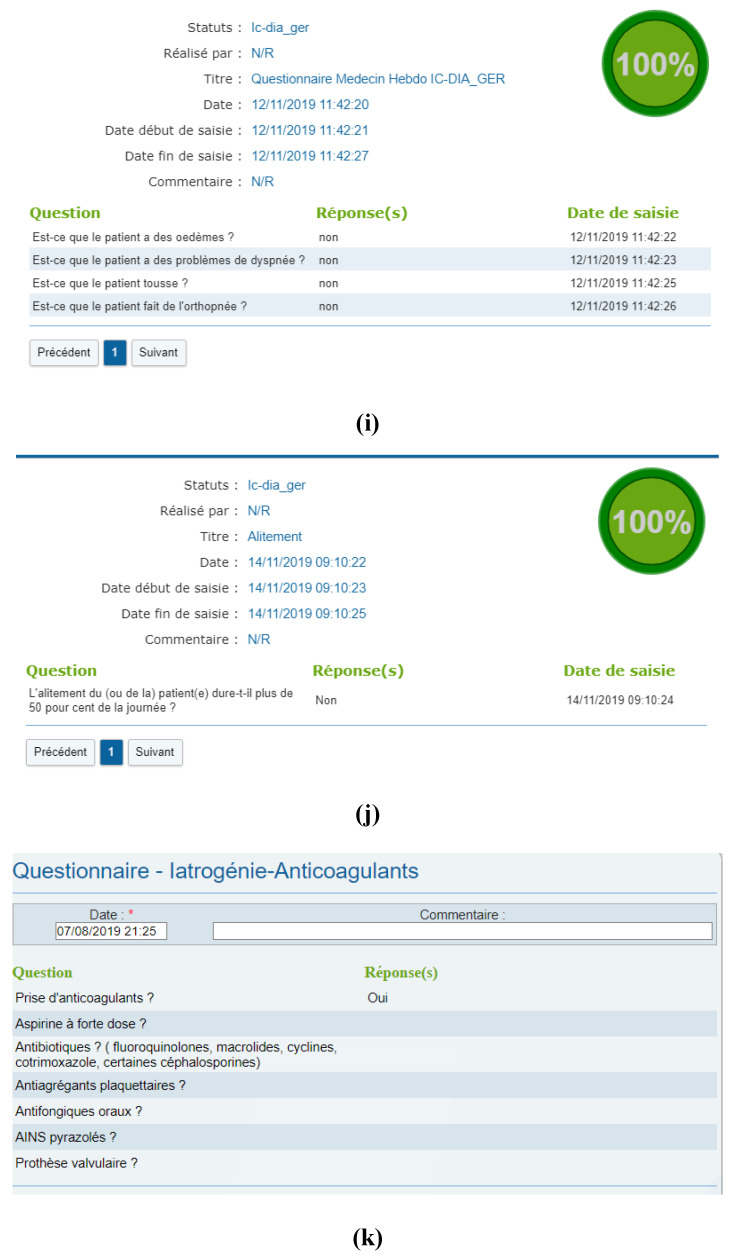

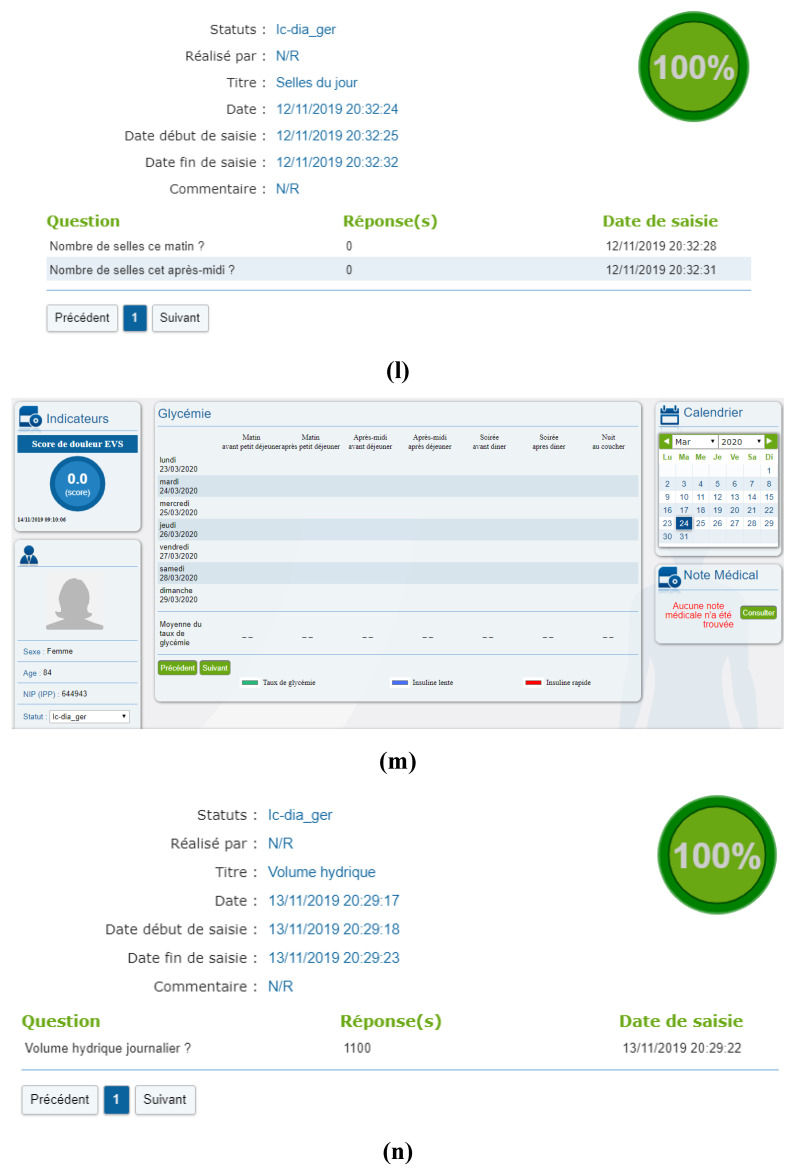
Measurements: (**a**) heart rate/oxygen saturation, (**b**) heart rate/oxygen saturation, (**c**) Illustration measure blood pressure, (**d**) blood pressure, (**e**) body temperature, (**f**) weight, (**g**) physical activity, (**h**) sleep quality, (**i**) heart failure questionnaire, (**j**) sleeping questionnaire, (**k**) anticoagulants questionnaire, (**l**) stools, (**m**) capillary glucose table, and (**n**) hydration questionnaire. (Source: PrediMed, Schiltigheim, France).

In the event of a consequent variation in vital signs (Figure 6), an alert is sent to the MyPredi platform.

## 8. Discussion

With the GER-e-TEC project, we can now offer medical teams and paramedics a remote monitoring device that optimizes the management of patients with geriatric syndromes and their accompanying conditions. This remote monitoring system will allow monitored patients to record their daily physiological data (weight, oxygen saturation, blood pressure, heart rate, blood sugar) and complete questionnaires on the state of their health. The data will be automatically sent to our MyPredi platform, which is designed to predict situations that are at risk of deteriorating.

A coordination unit will then be able to monitor the patient remotely, providing comprehensive and personalized treatment of the areas of concern detected by the platform and helping the patient with their therapy.

Patients monitored remotely:

- Physiological data analyzed daily;
- Help with therapy-related questions;
- Remote consultations.

Coordination unit:

- Processing of alerts;
- Help with therapy;
- Coordination of doctors and patients;
- Sending of reports to doctors on the health status of their patients;
- Monitoring of patient compliance.

Medical team:

- Medical advice if needed.

**Figure 7:**
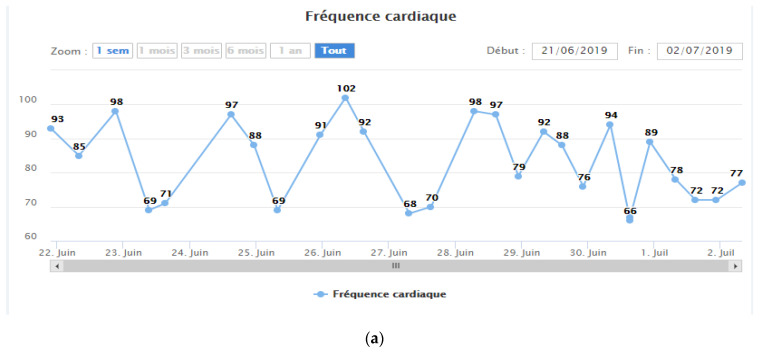

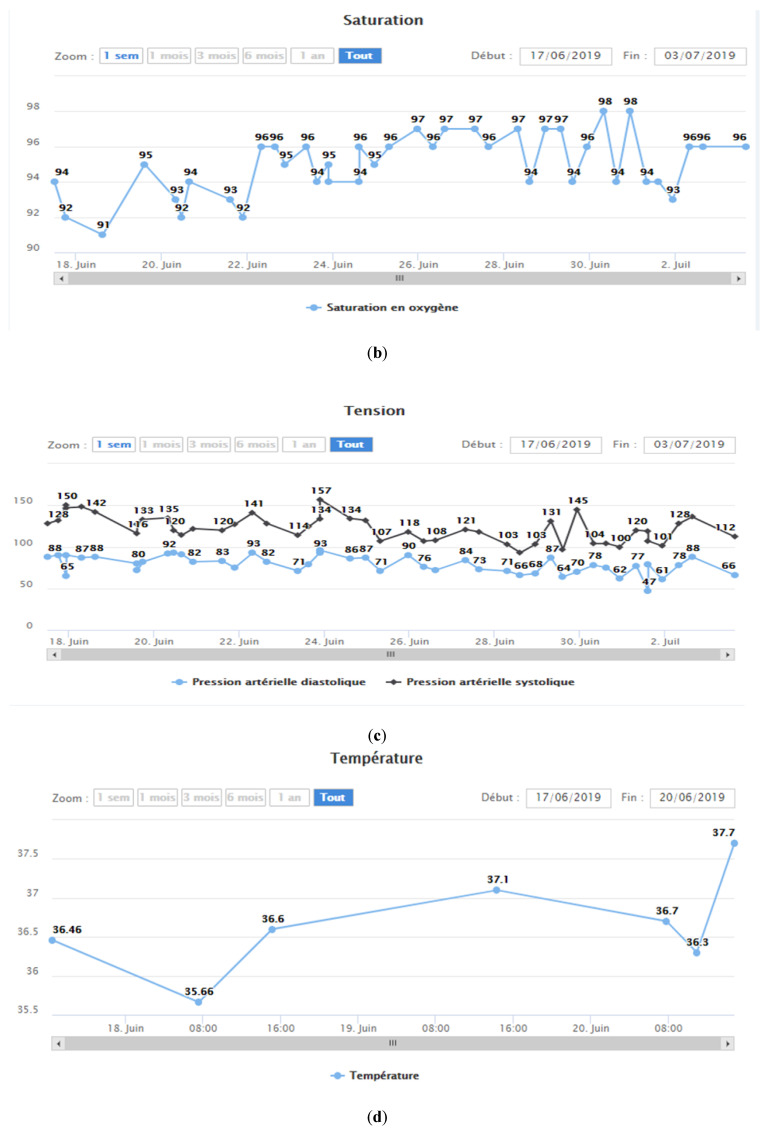

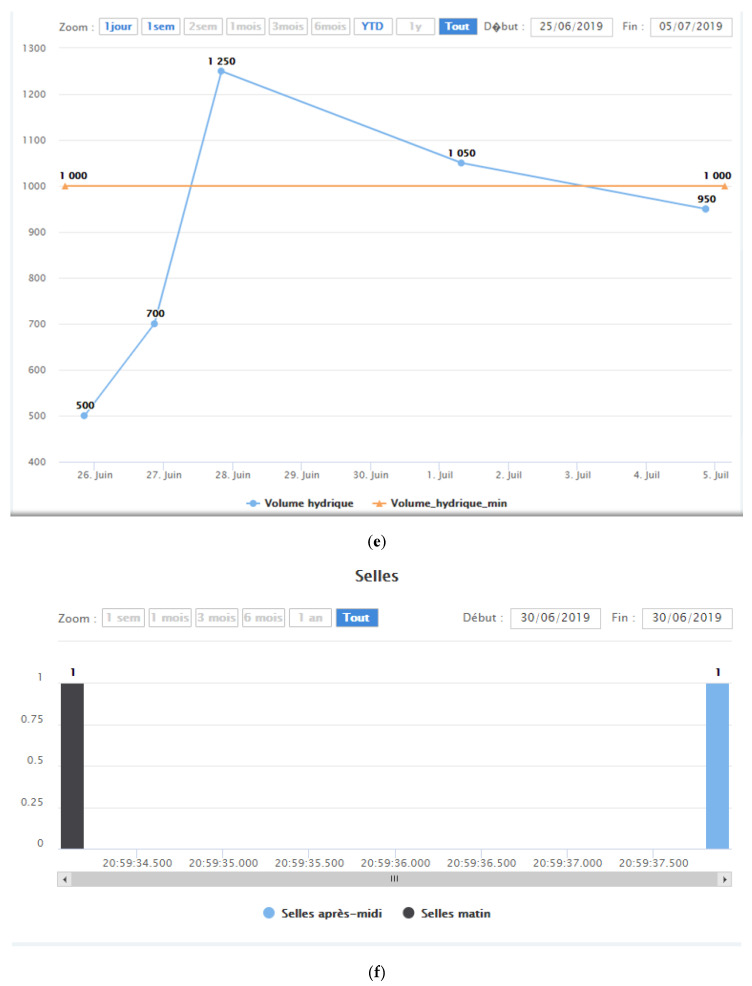

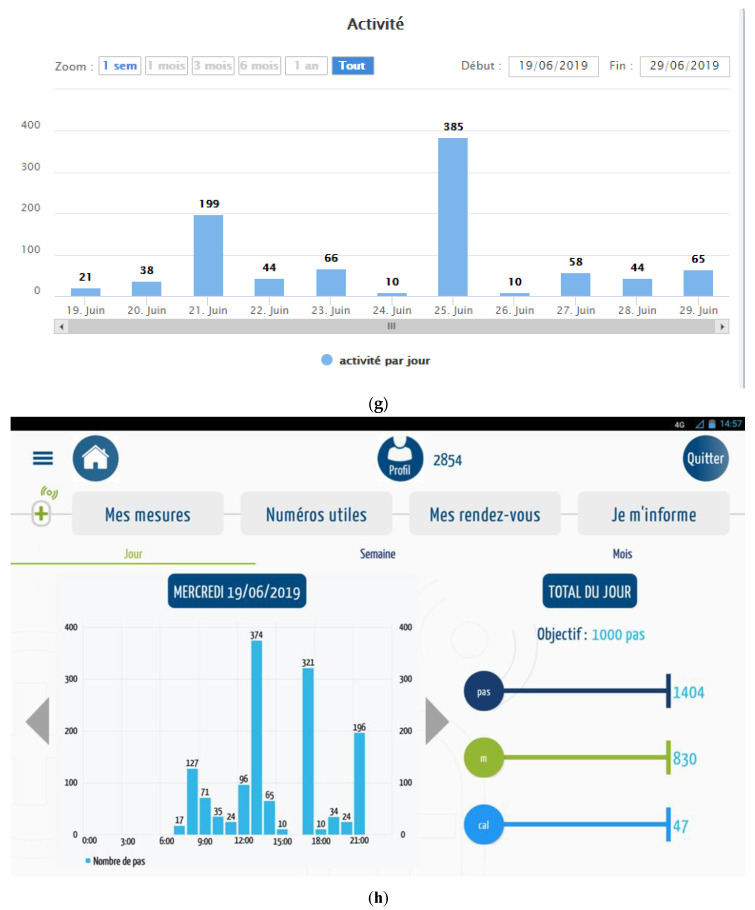

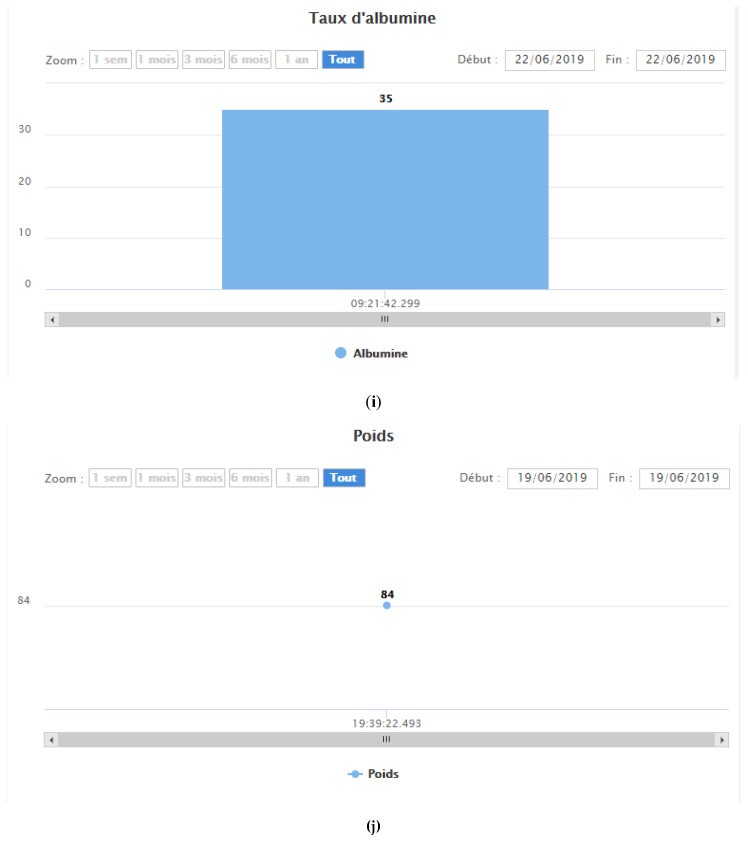

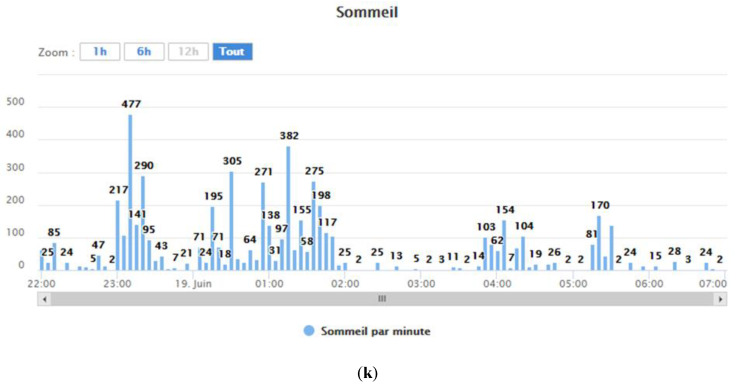
Daily monitoring measurements: (**a**) heart rate, (**b**) oxygen saturation, (**c**) blood pressure, (**d**) body temperature, (**e**) hydratation state, (**f**) Stools, (**g**,**h**) daily physical activity surveillance, (**i**,**j**) nutritional surveillance, (**k**) sleeping surveillance. (Source: PrediMed, Schiltigheim, France).

The GER-e-TEC project was tested on a patient (Figure 7) who, after providing written consent, was monitored daily by a team of healthcare professionals. The patient was given a pedometer to monitor his sleep and activity. The goal was to test the ergonomics and functionality of the remote monitoring platform on a daily basis before using it in the study.

**Figure 8:**
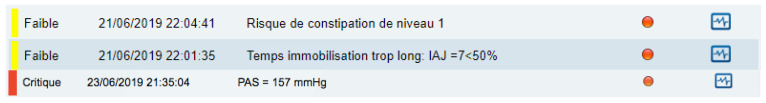
Alerts: faible/weak constipation risk (level 1), faible/weak inappropriate physical activity, and critique/critical systolic blood pressure = 157 mmHg. (Source: PrediMed, Schiltigheim, France).

By monitoring the patient’s geriatric health indicators and hemodynamic data, the remote monitoring system was able to issue alerts (Figure 8) with varying degrees of severity (low to critical).

This remote monitoring system can generate automated, non-intrusive alerts in the event of high-risk situations.

The feedback from healthcare professionals who used the MyPredi platform daily was positive. Surveys completed by nurses, nurses’ aides, doctors, and the patient found the satisfaction rate to be upwards of 90%. Thanks to the comments and suggestions from those who participated in the test, we have been able to make improvements to the platform interface both on the app and the web. The test helped us validate our choice of technology, as well as consolidate and assess the durability of the monitoring system. This proof of concept allowed us to test the remote monitoring system, evaluate its ergonomics, and analyze its use by the hospital care team.

The system will now be tested over the course of three months in an 18-bed medical unit at a University Hospital of Strasbourg. Between 100 and 110 patients will participate in the study using remote monitoring. This study will make it possible to consolidate the technological and ergonomic aspects of the system, and thanks to its daily use by doctors and nurses, allow for the MyPredi platform technology to be validated before the system is used in nursing homes (GER-IA project). The results of the study will be published upon completion.

## 9. Conclusions

Remote monitoring in nursing homes is a major issue in light of the current COVID-19 health crisis and the repeated (yet avoidable) hospitalizations required for the elderly. The goal of our “GER-e-TEC” project is to identify the early warning signs of (1) an exacerbation of geriatric problems (falls, constipation, dehydration, confusion, iatrogenesis, infections, bedsores, malnutrition), and (2) an exacerbation of accompanying syndromes (heart failure, diabetes, hypertension), and to provide for the early treatment of these conditions.

The initial phase of our project will consist of implementing and validating our technological processes in a Department of Internal and Geriatric Medicine at a University Hospital in Strasbourg (preliminary study (proof of concept)). This phase will involve around 110 elderly hospitalized patients and will allow us to validate our medical, technological, and ergonomic choices with elderly patients, healthcare professionals, and patient associations. In addition, our platform will provide healthcare professionals and paramedics with regularly updated geriatric information on each patient, i.e., their anthropometric, nutritional, cognitive, and iatrogenic data, and present a comprehensive digital overview of a standardized geriatric assessment that is both quick and easy to complete.

Once validated, this platform will be expanded to the nursing homes of the University Hospitals of Strasbourg.

## Figures and Tables

**Table 1:** Technical characteristics of the MyPredi platform sensors.

Sensors	Characteristics
**Balance**	A&D Medical, Model: UC-352BLE Bluetooth: 4.0
**Sphygmomanometer**	A&D Medical Model: UA-651BLE Bluetooth 4.0
**Pulse oximeter**	Jumper Model: JPD-500F Bluetooth: 4.0
**Pedometer**	Ecare Fit No model Bluetooth 4.0
**Glucometer**	FORA Advanced pro GD40 Model: TD-4272H/GD40h Bluetooth 4.0
**Thermometer**	Jumper Model JPD-FR302 Bluetooth 4.0
